# The role of civil society in strengthening intercultural maternal health care in local health facilities: Puno, Peru

**DOI:** 10.3402/gha.v9.33355

**Published:** 2016-12-15

**Authors:** Jeannie Samuel

**Affiliations:** School of Health Studies, Faculty of Health Sciences, University of Western Ontario, London, ON, Canada

**Keywords:** gender, indigenous, social accountability, human rights-based approach, health systems

## Abstract

**Background and objective:**

Peru's Ministry of Health has made efforts to increase the cultural inclusiveness of maternal health services. In 2005, the Ministry adopted an intercultural birthing policy (IBP) that authorizes and encourages the use of culturally acceptable birthing practices in government-run health facilities. However, studies suggest that indigenous women may receive inconsistent benefits from these kinds of policies. This article examines whether a grassroots accountability initiative based on citizen monitoring of local health facilities by indigenous women can help to promote the objectives of the IBP and improve intercultural maternal health care.

**Design:**

Findings are drawn from a larger qualitative research study completed in 2015 that included fieldwork done between 2010 and 2011. Semi-structured interviews were conducted with 23 women working as citizen monitors in local health facilities in Puno and 30 key informants, including frontline health workers, health officials, and civil society actors in Puno and Lima, and human rights lawyers from the *Defensoría del Pueblo* Office in Puno.

**Results:**

Monitors confirmed from their own personal experiences in the 1990s and early 2000s that respect for intercultural aspects of maternal health care, including traditional indigenous birthing practices, were not readily accepted in publicly funded health facilities. It was also common for indigenous women to face discrimination when seeking health service provided by the state. Although the government's adoption of the IBP in 2005 was a positive step, considerable efforts are still needed to ensure high-quality, culturally appropriate maternal health care is consistently available in local health facilities.

**Conclusions:**

Despite important progress in the past two decades, policies aimed at improving intercultural maternal health care are unevenly implemented in local health facilities. Civil society, in particular indigenous women themselves, can play an important role in holding the state accountable for quality care.

## Introduction

### Cultural exclusion in maternal health care in Peru

Although Peru has made important progress in reducing maternal mortality in recent decades, national figures mask deep inequalities in maternal mortality that exist within the country. The contrast is especially stark when comparing Andean and Amazonian regions with Lima, the national capital. For example, in the southern Andean department of Puno where this study takes place, the maternal mortality ratio in 2000 was estimated as 361 maternal deaths per 100,000 live births, nearly seven times the figure of 52 maternal deaths per 100,000 live births in Lima (1). Since obstetric emergencies remain a leading cause of maternal deaths in Peru, the fact that the percentage of institutional births is lower in both the Andean and the Amazonian regions is particularly concerning (2). Along with factors such as distance to health facilities, studies have identified cultural obstacles (e.g. health providers’ uneven knowledge and acceptance of traditional indigenous birthing practices, a lack of health providers with indigenous language skills, and discrimination and abuse within health facilities) as significant barriers to institutional delivery faced by indigenous women (1, 3). Birthing at home is still an important and positive option for many women, in particular those with low-risk pregnancies. Ideally, this would take place with the assistance of a skilled birth attendant (SBA), although this is not always possible. Nonetheless, access to high-quality, culturally appropriate maternal health care has a direct impact on the health rights of indigenous women in the country (4).

Inequalities in access to high-quality maternal health care services for indigenous women in Peru are part of a broader context of inequality in the country. In Peru, inequality has deep ethnic and racialized dimensions (5–7). For much of the country's history, Peru was divided ethnically and geographically between a Spanish-descended coastal population that dominated government and the economy, and an indigenous population that was largely tied to the land and engaged in subsistence agriculture or pastoralism in the Andes and traditional ways of life in the Amazon. Land reform in the 1960s and 1970s and large-scale migration of Andean populations to coastal cities and shanty towns in the decades that followed have complicated, but not changed the basic nature of these racialized divides. Drinot (5) argued that racism ‘is central to the exclusionary character of nation-building in Peru’ and that its attitudes are ‘institutional, hegemonic, legitimising, normalised’. Gender, ethnicity, and indigeneity have a complex relationship in the Peruvian Andes where women not only experience sexism but are often seen as ‘more indigenous’ than men and suffer, as a result, from unique and more profound social inequalities (8).

Important inroads have been made over the past two decades in terms of efforts to make health facilities more inclusive. Through the *Seguro Integral de Salud* (SIS) program, the government has extended free health coverage for a wide range of health services to the poorest segment of the population, many of whom are indigenous (9). A key step forward for maternal health care in Peru was made in 2005 when the Ministry of Health (MoH), in the wake of sustained civil society advocacy around the issue, passed a ministerial resolution adopting an intercultural birthing policy (IBP) that provides for the cultural accommodation of indigenous birthing practices. For the first time, this set out binding administrative rules that authorize MoH staff to allow and support women in state-financed health facilities to give birth according to indigenous cultural practices. The IBP changed the clinical birth protocol to allow for vertical birth positions, the use of warm clothes, heaters to ensure a warm environment, beds with wooden frames, and traditional food, drinks, and herbs (10). The policy also permitted mothers to have more than one person present to support them during the birth.

Although the adoption of the IBP has been an important policy change, studies of its effectiveness in practice suggest mixed results. For example, a 2009 investigation by Nureña found that compliance with the IBP at a national level was uneven (11). More recently, in a study of the perspectives of SBAs, Guerra-Reyes found that SBAs are provided with only weak institutional support to implement the IBP (12).

Institutional discrimination and marginalization may be continuing to curtail the effectiveness of the IBP (11). Thorp and Paredes argued that the inequalities tied to multiple axes of oppression in Peru are able to persist across time and despite changes in law and policy in part because they are embedded in the country's institutions, including the health care system (13). Ideas regarding racialized and gendered hierarchies can shape both the private attitudes and public policies that influence the design, administration, funding, and implementation of health care services. In addition, health systems for marginalized populations, such as Peru's state-funded system serving the poorest segment of society, are often underfunded, poorly managed, and may subject health workers to difficult and disrespectful work conditions (14). As Freedman and Kruk observed, in these circumstances ‘providers’ professional ideals often succumb to the pressure of emotional and physical survival strategies – a midwife providing compassionate care at one moment might be overwhelmed by the stress of unmeetable demands in the next and lash out at the women she attends’ (15). These factors present particular challenges to the implementation of policies designed to combat social, including cultural, exclusion in service delivery.

### The citizen monitoring initiative in Puno

An initiative in Puno, Peru has been working to strengthen the quality of health care services including maternal health care and adherence to the IBP through the promotion of citizen-led accountability in local health facilities. The initiative was initially conceived of as a pilot activity by actors from the NGOs CARE Peru, CARE International, and Physicians for Human Rights in 2008. It expanded over time and was supported by CARE Peru and ForoSalud, the country's leading civil society network promoting health rights. The initiative recruited, trained, and supported Quechua speaking indigenous women from community-based organizations (CBOs) in the department of Puno to act as volunteer citizen monitors to observe and report on the delivery of health care services in their local publicly provided facilities. Lawyers from the Puno office of the *Defensoría del Pueblo*, Peru's National Human Rights Ombudsman's Office, also provided the monitors with training and support, as did other strategic allies. The initiative uses a human rights-based approach to health to promote local level accountability of district health facilities. Direct NGO assistance to the original initiative ceased in 2013, but monitors continue their activities through their local CBOs. Former members of the project continue to provide technical assistance on a volunteer basis (16, 17).

This raises the question whether grassroots accountability initiatives such as citizen monitoring can help to promote the objectives of policy changes aimed at improving intercultural maternal health care. Grassroots and citizen-led initiatives face a significant challenge. They rely on marginalized actors such as indigenous women to play a significant role in influencing practice within the health care system. If the marginalization of indigenous people within the health care system is part of the problem, how are these actors able to influence health care practices?

## Methods

The findings presented here are drawn from a larger qualitative research study that included fieldwork conducted in 2010 and 2011 by the author and two research assistants from Puno (17). Methodologically, this study used an institutional ethnographic approach to examine the work of citizen monitors in Puno, Peru. Institutional ethnography is based on the premise that analyzing the work processes and other experiences of a particular group of people can provide an important vantage point to understand a broader set of social and institutional relations (19). The author uses the notions of work processes and work knowledge to help explore and understand the work, roles, and working relationships of the citizen monitors in Puno. This involves an in-depth examination of the daily monitoring work done by this group of women to promote change in reproductive health service delivery. This approach is well suited to gain insight into the complex power relations that shape the monitors’ unequal engagement with their local health facilities.

Data were collected by the author and the two assistants using a purposive sample of 53 semi-structured interviews. These included 23 initial interviews with women from two CBOs who volunteered as citizen monitors in eight local health facilities in the districts of Azángaro and Ayaviri in Puno. In subsequent rounds of data collection, 30 key informant interviews were conducted in Puno and Lima. Key informants included local health officials, frontline health workers, and civil society actors, as well as lawyers from the *Defensoría del Pueblo* (Peru's National Ombud's Office) and the regional office of the National Public Health Insurance Program. The interviewers digitally recorded all the data and also kept extensive written field notes. Data were transcribed verbatim in Spanish by the research assistants. The author repeatedly analyzed the transcripts section by section. Using an open coding process, an initial set of main codes was developed based on key themes that emerged from the data set. Each of these thematic categories was further analyzed to develop a set of sub-themes. In line with an institutional ethnographic approach, particular attention was given to themes that correspond to the work processes of the citizen monitors as well as how these processes are linked to broader institutional arrangements. Analysis of interview data was complemented by the use of key documents including grey literature. Once a series of main findings were identified, short quotes were drawn from the data set, where these effectively illustrated findings. The author translated these quotes into English. A sample of quotes drawn from the interviews was verified by a professional translator.

A series of steps were built into the research design in order to strengthen the trustworthiness of the data and interpretation. Local experts (scholars and practitioners) were consulted during the initial design phase of the research protocol. All interview guides were professionally translated and checked by experts from the region. The guides were pilot tested and adjusted accordingly. Interviews were conducted with a wide range of stakeholders occupying different social locations in order to understand multiple perspectives around common themes. During fieldwork and data analysis, the author engaged in periodic consultation with research assistants, key stakeholders, and university faculty advisers to examine her interpretation of the data.

Certain study limitations should be noted. The study is limited to one geographic location for reasons of time and resources. The decision was made to focus on interviewing and document review as data collection tools. Participant observation could have added further depth to the data set but was not feasible due to time and resources. Interviews were conducted with citizen monitors and other key stakeholders relevant to their work including health workers and health officials. Based on the limited scope of the study, a decision was made not to interview health users at this stage. It is hoped that a focus on health care users will be the focus of a future research project.

### Ethics considerations

This article draws on data and findings from a larger study that was the author's doctoral research through the University of Toronto. The study received ethical approval (25277) through the University of Toronto Research Ethics Board in 2010. Ethics approval was reviewed and renewed every year until completion of the study. The entry point for the research in Peru was through the main Peruvian NGO supporting the citizen monitoring project at that time. The organization had no formal internal ethical review process for research. In lieu of an internal ethics committee, the NGO's Health Team National Coordinator was asked to provide ethics comments on the research proposal. Based on this consultation, CARE Peru provided a letter of endorsement of the research that was included in the submission to the University of Toronto's Ethics Review Board.

## Results

### Citizen monitoring and implementation of the intercultural birthing policy

When the intervention began, monitors acknowledged that there had already been substantive improvements in terms of respect for intercultural birthing practices in their local health facilities since their earlier encounters in the 1990s and early 2000s (17), but they also noted that there was still much more to be done. Monitors reported that health workers did not always fully observe the IBP and that considerable efforts were still needed to promote culturally appropriate birthing practices and other health rights in these facilities. For example, in the words of one monitor:

I'd say that since around 2005 there were these rights… But it wasn't very solid. They hadn't fully succeeded putting these rights in practice. (Interview Anon.4, 2010)

Similarly, an organizational ally of the monitors noted that ‘an investigation (by a US-based NGO) had shown that there had been maternal deaths that shouldn't have happened, that they were linked to discrimination and mistreatment’ (Interview Anon.15, 2011).

Despite the uneven application of the IBP, citizen monitors suggested that the existence of the policy was still an important resource in their efforts to promote intercultural inclusion in their district health facilities. The monitors’ training on MoH policies and related laws laws allowed them to call upon ministerial policies like the IBP when advocating for a patient. One monitor observed how this knowledge changed their potential interactions in health facilities:

We were trained about rights …. That we can monitor authorities, the Ministry of Health, that we have the right to denounce them to the prosecutor. That's what we learned. (Interview Anon.3, 2010)

Another monitor reinforced this view concerning the importance of knowing about the relevant laws and policies:

We can get information, we can request a report from the authorities about something we want to know about and that we have doubts about…So the laws protect us, now that we know what they're about. (Interview Anon.11, 2010)

Monitors were also able to collaborate with lawyers from the regional *Defensoría del Pueblo* Office to support them when necessary. Technically, the *Defensoría* is charged with providing oversight of service delivery through Peru’s public institutions including the MoH, although it lacks the resources to do this consistently. Through its partnership with the citizen monitors, the *Defensoría* was able to exert a much more consistent influence within the monitored health facilities. As one monitor remarked:

The *Defensoría* helps us because when we call them about very serious cases that are not getting seen quickly, they make sure that these get attention. They call the Director of the hospital … This is something that we're able to do as monitors. (Interview Anon.3, 2010)

A staff member from the *Defensoría's* office acknowledged the importance of the monitors’ presence in helping to promote compliance with the IBP and other ministerial rules designed to promote culturally respectful practices:

We feel like a positive thing from this citizen monitoring is that treatment by the doctors, the nurses, the midwives has been changing. For example, in Azángaro around intercultural issues, the health workers were notorious. If women from rural areas came with their cloth and fleece to receive their newborn, they [the health workers] would reject it, saying ‘No, you have to buy these other things here, like these pampers [disposable diapers]’. And often people didn't like that. But I think with monitoring they're [health workers] starting to get better. (Interview Anon.23, 2011)

Monitors also have become important advocates outside of the monitoring process. For example, a lawyer from the *Defensoría* noted that monitors intervened as a group when the municipality was going to buy equipment for a new birthing center. The monitors lobbied successfully to have beds purchased with wooden rather than metal frames, in line with both Andean birthing customs and guidelines from the national MoH's IBP. One lawyer from the *Defensoría* observed:

They don't just participate in the monitoring spaces …. There was a budget for the Birthing Centre and the municipality was going to buy equipment. The monitors intervened as an organization. They said, ‘We don't want these types of beds, these frames made of iron. We want these made of wood, which are consistent with our customs’. They made all sorts of other changes too. I think it's important, this theme of articulation, this strengthening of an organization. That it's articulated with other state institutions, like the municipality for example. (Interview Anon.24, 2011)

In another instance, monitors supported doctors’ requests in a town meeting for funds from their municipality to buy a new ambulance for the hospital. One doctor recounted the assistance he received from the monitors at a municipal meeting:

When the Ministry of Health makes a request or a demand at a municipal meeting to all of civil society, the citizen monitors are our witnesses. They're the ones who confirm our needs or who confirm the quality of attention that we've managed or not managed to provide. For us it's a huge support. Personally, to me, the monitors are a huge support. For example, when we've made a request and the municipality wonders if we need this money, the monitors have helped us …. Due to their participation, since they are the [health care] users, in the end they're the ones who are believed. The general population listens more to them in these meetings than to us. (Interview Anon.29, 2011)

The doctor explained the important role played by the civil society monitors in giving legitimacy to this request for funds. An additional ambulance can be lifesaving for rural women facing obstetric emergencies.

Some real progress has been made in relation to advancing maternal and reproductive health rights among marginalized women since the beginning of the monitoring process. However, several monitors also noted that improvements are inconsistent. Complaints concerning patient mistreatment continue to be high. Charges for services that should be covered by public health insurance remain a consistent problem (14). For example, one monitor noted it is not uncommon ‘to try to charge you for your bed when you deliver’ (Interview Anon.3, 2010). The monitors acknowledge that health users tell them that treatment is much better when the monitors are present, but that it often slides back to old patterns when the women are no longer on their shifts. ‘Now, when we go [to monitor], treatment is good. But then when we're absent, it always goes down somewhat’, remarked one monitor (Interview Anon.8, 2010). This suggests that the impact of monitoring is very much a work in progress and that additional measures are potentially needed to embed these new patterns of behavior more deeply within the health care system. As one of the monitors explained, ‘our idea is that with or without monitoring, treatment should be good, this is what we think. This is our goal’ (Interview Anon.8, 2010).

### The struggle to monitor

The women who volunteer as citizen monitors in this initiative are Quechua speaking members of local communities close to the targeted health facilities. They all have extensive leadership experience in various community organizations. Many had previously served as reproductive health promoters in a previous NGO-supported project. As a result, they were well acquainted with the workings of the health system. All the monitors received training from the supporting allied organizations prior to beginning the citizen-led accountability process. Nevertheless, their early days of monitoring proved challenging. Monitors were often challenged by health workers when they first attempted to carry out their monitoring activities. One monitor described her early experience:

I didn't want to go again after the first time. They [health workers] treated me badly. I didn't want to go back. Even my husband told me, ‘This is going to make you feel badly’. I felt they had lowered my morale, me who had such high self-esteem, they lowered it to the ground. (Interview Anon.6, 2010)

Monitors pointed to a combination of factors that helped them to establish the authority to monitor local health care facilities. Monitors are trained in relevant laws and policies concerning health and human rights. They refer with pride to their ability to cite the laws and ministerial resolutions that support their monitoring activities. In the words of one monitor: ‘So the laws protect us, now that we know what they're about … Even today the authorities don't know, they don't know about these laws’ (Interview Anon.11, 2010). The initiative's allied organizations, including the *Defensoría del Pueblo*, also issued special badges that the monitors wear when on duty.

Strictly, monitors are supposed to observe and document what takes place in the health facility for follow-up action. However, in practice, many monitors choose to intervene in serious matters as they are taking place. In these cases, monitors may call lawyers from the *Defensoría* or officials from SIS. The ability of monitors to mobilize these more powerful allies appears to translate into increased personal influence in health facilities over time (17). Monitors need to continuously struggle to establish recognition and authority within the health care facilities. This is a challenge, but both monitors and key informants report that this has improved over time as the health workers become accustomed to the monitors’ presence.

As part of the monitoring program, monitors and their organizational allies attend regular meetings with district health officials to present summaries of their findings. This allows monitors to raise questions and concerns with senior administrators. This can translate into increased influence in health care facilities. However, this mechanism is still fragile. Instability within the regional health directorate and the frequent turnover of high-level health officials frequently lead to delay or cancellation of these official meetings (17).

On balance, results from the study reveal positive gains at a facility level through citizen monitoring. However as already noted, monitors acknowledged that these improvements are inconsistent.

## Discussion

The marginalization of indigenous women in health care facilities is a product of a broader set of normalized power relations that take place both inside and outside of health facilities. Discrimination, cultural exclusion, and abuse within a health facility are potential outcomes of these power relations (19). The existence of a ministerial policy such as the IBP is important but will not necessarily change these dynamics within the health care setting. Policies need to be championed to be brought into practice. If this is not being effectively or fully done within the MoH bureaucracy, then the task falls to civil society. However, groups facing multiple axes of oppression face the greatest challenges when attempting to exercise this kind of influence. This highlights an important contradiction: members of marginalized groups are least able to rely on the policies designed to address their marginalization.

This is particularly important, given that, as many scholars have noted, interculturality in health is in essence about navigating power relationships (20–22). As Carmen Yon argued in her introduction to a recent team-led case study related to intercultural health issues in Peru, and drawing on the work of other key scholars in the field, the discussion on interculturality needs to include and also move beyond a focus on issues such as indigenous language use or respectful treatment. She noted that interculturality in health requires questioning social relationships and hierarchies between professionals and health users, as well as unequal relationships between biomedical systems and indigenous health systems (20).

The citizen monitoring initiative in Puno is one example of civil society efforts, in this case, spearheaded by indigenous women, to challenge entrenched biomedical practices and push for more consistent provision of accessible, high-quality intercultural maternal health services directly within local health facilities. While imperfect, the relative success of the citizen monitoring initiative in Puno appears to stem from its ability to influence power relations at least at the micro level of the health facility, through the direct presence and actions of citizen monitors and their collaboration with strategic allies. These monitors are able to exercise authority in certain circumstances and help to insist on the application of policies such as the IBP.

However, this ability to exercise authority is precarious and contingent. The influence of monitors also has clear limits. Some problems in health care facilities are caused or exacerbated by systemic deficiencies and neglect within the health care system serving the poor (16). These more entrenched issues related to high-quality intercultural maternal service provision remain difficult for this type of civil society initiative to directly tackle through its micro interventions at a facility level.

## Conclusions

In conclusion, policies designed to combat historically entrenched discrimination and cultural exclusion for women facing multiple oppressions may require special accountability mechanisms to be effective. The key lesson from the citizen monitoring project in Puno is that such mechanisms must be able to influence power relations within the spaces they are intended to affect. For marginalized and indigenous women, this is an uphill struggle that will require multiple strategies tailored to the particular circumstances in question. The discussion in this article suggests that frontline, facility-based citizen monitors, with direct links and experience with these lived realities along with direct links to strategic allies, can play a crucial role in beginning to challenge these power dynamics and can serve as a channel to further foster the rights of women facing multiple forms of oppression.

## Paper context

The failure of health systems to accept traditional birthing practices can be a significant barrier to institutional delivery for indigenous women. However, policies designed to encourage culturally inclusive birthing practices in health facilities may have limited effectiveness where discrimination is systemic. By exploring a grassroots, human rights-based citizen monitoring initiative in Andean Peru, this article shows that civil society accountability mechanisms can play a role in supporting policies in favor of intercultural birthing practices.
